# Non-linear Heart Rate Variability as a Discriminator of Internalizing Psychopathology and Negative Affect in Children With Internalizing Problems and Healthy Controls

**DOI:** 10.3389/fphys.2018.00561

**Published:** 2018-05-23

**Authors:** Charlotte Fiskum, Tonje G. Andersen, Xavier Bornas, Per M. Aslaksen, Magne A. Flaten, Karl Jacobsen

**Affiliations:** ^1^Department of Psychology, Norwegian University of Science and Technology, Trondheim, Norway; ^2^Department of Psychology, University of the Balearic Islands, Palma, Spain; ^3^Department of Psychology, The Arctic University of Norway, Tromsø, Norway

**Keywords:** non-linear time-series analysis, HRV, sample entropy, RMSSD, detrended fluctuation analysis, pre-ejection period, anxiety, depression

## Abstract

**Background:** Internalizing psychopathology and dysregulated negative affect are characterized by dysregulation in the autonomic nervous system and reduced heart rate variability (HRV) due to increases in sympathetic activity alongside reduced vagal tone. The neurovisceral system is however, a complex nonlinear system, and nonlinear indices related to psychopathology are so far less studied in children. Essential nonlinear properties of a system can be found in two main domains: the informational domain and the invariant domain. sample entropy (SampEn) is a much-used method from the informational domain, while detrended fluctuation analysis (DFA) represents a widely-used method from the invariant domain. To see if nonlinear HRV can provide information beyond linear indices of autonomic activation, this study investigated SampEn and DFA as discriminators of internalizing psychopathology and negative affect alongside measures of vagally-mediated HRV and sympathetic activation.

**Material and Methods:** Thirty-Two children with internalizing difficulties and 25 healthy controls (aged 9–13) were assessed with the Child Behavior Checklist and the Early Adolescent Temperament Questionnaire, Revised, giving an estimate of internalizing psychopathology, negative affect and effortful control, a protective factor against psychopathology. Five minute electrocardiogram and impedance cardiography recordings were collected during a resting baseline, giving estimates of SampEn, DFA short-term scaling exponent α_1_, root mean square of successive differences (RMSSD), and pre-ejection period (PEP). Between-group differences and correlations were assessed with parametric and non-parametric tests, and the relationships between cardiac variables, psychopathology and negative affect were assessed using generalized linear modeling.

**Results:** SampEn and DFA were not significantly different between the groups. SampEn was weakly negatively related to heart rate (HR) in the controls, while DFA was moderately negatively related to RMSSD in both groups, and moderately positively related to HR in the clinical sample. SampEn was significantly associated with internalizing psychopathology and negative affect. DFA was significantly related to internalizing psychopathology.

**Conclusions:** Higher invariant self-similarity was linked to less psychopathology. Higher informational entropy was related to less psychopathology and less negative affect, and may provide an index of the organizational flexibility of the neurovisceral system.

## Introduction

Internalizing psychopathologies like anxiety and depression are among the costliest and most prevalent disorders in society (Pincus and Pettit, 2001; Kessler, 2012). One of the reasons for this is the early debut (Kessler and Walters, 1998; Kessler et al., 2007) and high risk of carrying a psychiatric disorder into adulthood (Pine et al., 1998; Pine, 1999; Kessler, 2012). Dysregulated negative affect, the tendency to repeatedly experience negative affect and distress (Watson et al., 1994), may be a common component in both anxiety and depression and can increase the risk of internalizing psychopathology (Watson et al., 1995). Not only are anxiety and depression risk factors for later psychopathology and suicide (Beesdo et al., 2009; Kessler, 2012; Maughan et al., 2013), but they may also increase the likelihood of later cardiovascular disease and mortality through autonomic nervous system (ANS) dysregulation (Chalmers et al., 2014; Koenig et al., 2016; Kemp et al., 2017).

The neurovisceral integration model (Thayer and Lane, 2000, 2009) states that internalizing psychopathology and dysregulated negative affect is characterized by an imbalance between positive and negative feedback loops in the neurocardiac system and the central autonomic network, an internal regulatory network consisting of structures like the amygdala, hypothalamus, nucleus of the tractus solitarius and the insular cortex (Benarroch, 1993). In this model stressful internal or external stimuli lead to hypoactivation of the frontal cortex, resulting in vagal withdrawal. As the vagal nerve ordinarily works as a brake on sympathetic activation, this withdrawal readies the organism for threat through increased sympathetic nervous system (SNS) activation. In the short term, and in the face of actual threat, this reaction leads to adaptation to environmental demands and increased chances of survival. In the case of anxiety or emotional dysregulation, a chronic disinhibition of the SNS in the face of non-threatening stimuli instead leads to difficulties with adaptive and flexible behavior as well as health problems (Thayer and Friedman, 2004).

Dysregulation of affect, anxiety and depression is associated with dysregulation in the ANS as indexed by a loss of heart rate variability (HRV), in both adult (Kemp et al., 2010; Chalmers et al., 2014) and child (Koenig et al., 2016; Paniccia et al., 2017) populations. Therefore, HRV has been proposed as a general biomarker of psychopathology and dysregulated negative affect (Beauchaine and Thayer, 2015). So far, most research on HRV is based on methodology calculating variability in the time- or frequency domain, giving a measure of average or magnitude values for variability using linear approaches to signal analysis (Laborde et al., 2017). The past decades have seen an increase in more mathematically-sophisticated nonlinear methods for cardiac analysis (Sassi et al., 2015). This is important for several reasons.

First, complex adaptive systems exhibit features of high organization combined with the capability for high input-driven variability to allow for a balance between predictability of behavior and flexible adaptability (Tyukin, 2011). There is general agreement that the aim of the neurocardiac system is to support flexible and adaptive interaction with the environment while balancing internal and environmental demands, not least in the social domain (Porges, 1995; Thayer and Lane, 2000; Beauchaine, 2001). Complex adaptive systems dependent on, and interacting with, external factors typically exhibit nonlinear feedback processes (Tyukin, 2011).

Second, apart from being embedded in a complex context, the human neurocardiac system is also a complex physiological system. The heartbeat is influenced by the central autonomic network, SNS and parasympathetic nervous system (PNS) activation, baroreflexes, respiration and frontal influence, as well as highly interconnected neurons in the heart (Randall et al., 1996; Berntson et al., 2007; Wake and Brack, 2016). The subsystems affecting the heartbeat are reciprocally interacting through positive (excitatory) and negative (inhibitory) feedback loops, and afferent and efferent connections. The presence of multiple reciprocally-interacting feedback loops across different levels (e.g., heart, autonomic and central nervous systems, internal, external) gives rise to nonlinear processes (Glass and Mackey, 1988; Bertuglia and Vaio, 2005; Voss et al., 2009). Nonlinear systems and processes cannot be captured properly using only linear methodology (Glass and Mackey, 1988; Bertuglia and Vaio, 2005; West, 2014) and a nonlinear approach should be taken for cardiac analysis (Young and Benton, 2015).

Essential nonlinear properties of any system can be found in five main domains. These are the following: invariant (analysis of fractal or scale-free self-similarity), informational (entropy-methods), statistical (for instance, symbolic dynamics), geometrical (methods like Poincaré or recurrence plots) and energetic (for example time-irreversibility measures) (Voss et al., 2009; Bravi et al., 2011; Sassi et al., 2015). According to a 2015 consensus-report on nonlinear HRV, the invariant and informational domains represent the most promising areas to investigate (Sassi et al., 2015). Both have also been shown to relate to internalizing psychopathology (de la Torre-Luque et al., 2016).

detrended fluctuation analysis (DFA) is one of the most widely-used methods within the invariant domain. Through DFA the scale-invariant self-similarity, or fractal properties, of a time series can be established (Goldberger et al., 2002). A system high in self-similarity shows the same patterns of organization at different scales of magnification. Fractals are ubiquitous in biological systems, perhaps the best-known examples being those of branching in trees, the arteries of the body or the pulmonary system. A time series can also be analyzed for self-similar correlations over shorter and longer intervals to establish scale-invariant properties. The healthy heartbeat time series shows scale-invariant self-similarity, and a loss of self-similarity in the cardiac time series occurs in disease and aging (Goldberger et al., 2002; Lipsitz, 2004; Perkiömäki, 2011; Sassi et al., 2015).

The informational domain includes specific algorithms to estimate the entropy in a given time series (Bravi et al., 2011; Sassi et al., 2015). Entropy refers to the rate of new information a system generates as it evolves. Highly regular, predictable systems do not generate much new information, and are therefore characterized by low values of entropy. Complex systems, on the other hand, behave in more unpredictable ways, display irregular behavior, and show high entropy values. In trauma or disease the heartbeat generally shows a decrease in entropy consistent with a loss of information-based complexity, although sometimes high entropy rates can be a marker of pathological disorder as well, as seen in for instance heart arrhythmias (Costa et al., 2005).

Non-linear HRV-measures have shown promise as markers of internalizing psychopathology, generally linking lower information-based complexity, lower scale-invariant self-similarity, or less-ordered complexity to disorder (de la Torre-Luque et al., 2016). A recent review of HRV-research in children and adolescents related to internalizing psychopathology shows that studies using nonlinear methods still represent a small section of the published research (Paniccia et al., 2017).

This study aims to investigate the possible relationship between two key nonlinear cardiac properties, scale-invariant self-similarity and entropy, as they relate to internalizing psychopathology and negative affect in children with internalizing difficulties and healthy controls. We will use one measure of scale-invariant nonlinear HRV, short-term detrended fluctuation analysis (DFA-α_1_), and one metric from the information-based entropy domain, sample entropy (SampEn). To further knowledge about the clinical utility of nonlinear cardiac complexity we want to see if SampEn or DFA-α_1_ can provide information not inherent in more traditional HRV measures, by modeling them alongside the root mean square of successive differences (RMSSD), a measure of vagally-mediated high-frequency HRV. As the neurovisceral integration model describes dysregulation in the neurovisceral system as a disinhibition of SNS activity we also include pre-ejection period (PEP), a measure of sympathetically modulated contractility of the heart (Newlin and Levenson, 1979). Finally, we investigate the relationship between the nonlinear HRV measures and the subscales of the Child Behavior Checklist (CBCL) (Achenbach and Rescorla, 2001) related to internalizing psychopathology and the subscales in the Early Adolescent Temperament Questionnaire, Revised (EATQ-R) (Ellis and Rothbart, 2001) related to negative affect. Here we also include the Effortful Control scale from the EATQ-R as a measure that is linked to risk for psychopathology and frontal executive function (Nigg, 2006; Verstraeten et al., 2009).

### Hypotheses

H1: Nonlinear HRV should differ between the two groups.
H2: Higher nonlinear HRV should be related to less internalizing psychopathology, less negative affect and more effortful control.
H3: Nonlinear HRV, represented by SampEn and DFA-α_1_, should provide explanatory power beyond linear PNS and SNS measures.

## Materials and methods

### Sample and participant selection

The study was part of a larger study on child psychotherapy (also described in Fiskum et al., 2017) and had a between-groups design consisting of a clinical and a control group assessed with electrocardiography (ECG) and impedance cardiography (ICG), along with parental reports of emotional and clinical problems.

Forty children between 9 and 13 years were referred to the study from parents, mental health providers, child protective services, general practitioner doctors and school nurses. The children were selected for inclusion in the clinical group based on scores on the CBCL above the normal range in the internalizing spectrum (anxiety or depression). Four children were excluded due to normal CBCL scores.

The children were screened for medicine use and two children were excluded for the use of allergy and asthma medicine. Other exclusion criteria were severe learning difficulties and severe co-morbid mental or physical disorders. One child was excluded due to a congenital heart defect, and one child was excluded due to possible psychotic symptoms. The clinical group consisted of 32 children (16 girls). Mean age was 10.9 years (SD 1.3).

Twenty-eight children between 9 and 13 years were recruited to the control group mainly through information at parental meetings at schools in Trondheim. Eight children were recruited through convenience sampling. The inclusion criteria were normal range scores on the CBCL and no known history of contact with mental health services for any cause. In addition, the same exclusion criteria as for the clinical group were applied. One child was excluded due to a referral to mental health services for a possible developmental disorder, one child due to a clinical range CBCL score, and one child due to medicine use. The full control group consisted of 25 children (14 girls). Mean age was 10.3 years (SD 1.2). The groups did not differ significantly in age as measured with a Mann-Whitney *U*-test (*U* = 286, *p* = 0.067).

The children were recruited from Trondheim (47 children) and Oslo (10 children) and included 53 Caucasian children and one Asian (clinical group), one Middle Eastern (control group) and two African heritage children (one in each group). Thirty-five children lived in a two-parent household, 13 lived with a single parent, and 9 did not say. In the clinical group 24 mothers and 20 fathers had completed higher education (defined as more than 12 years of education), while this was the case for 21 mothers and 18 fathers in the control group. There were no statistical differences between the groups on gender balance (*U* = 376, *p* = 0.66), type of household (*U* = 255, *p* = 0.58), maternal (*U* = 307, *p* = 0.18) or paternal (*U* = 278, *p* = 0.30) education.

The participants and their parents were given oral and written explanations of the study and the procedure involved, and parents signed a written consent. The study was approved by the Norwegian Regional Committees for Medical and Health Research Ethics, and was in compliance with the Helsinki Declaration (World Medical Association, 2013). Anonymous data from the project will be uploaded to the Norwegian Centre for Research Data (www.nsd.uib.no) upon completion, likely at the end of 2020.

### Assessments and measures

#### The child behavior checklist

The Child Behavior Checklist 6-18 parent rating form (Achenbach and Rescorla, 2001) is a clinical rating scale giving an age- and gender-adjusted assessment of a wide array of mental health concerns. A total of 120 items describing problematic behavior is rated by a parent on a 3-point Likert scale, where 0 means that the item is *not true*, 1 means the item is *somewhat or sometimes true*, and 2 means it is *very often true / very true*.

The Internalizing Problems scale in the CBCL provides a scaled score of internalizing psychopathology based on the total problematic behavior reported along the dimensions of anxiety, affective problems and somatization, and reflects the child's total dimensional load on common childhood internalizing spectrum mental disorders. The CBCL is a significant predictor of later disturbance in children between 4 and 11 years over an eight-year (Ferdinand and Verhulst, 1995) and 14-year span (Hofstra et al., 2002), and corresponds well with clinical judgment of psychopathology by health-care professionals (Verhulst and van der Ende, 1991). In the present study we focused on the Internalizing Problems scale (33 items), as well as two clinical scales related to internalizing disorder: Affective Problems (13 items, Cronbach's α = 0.83) and Anxiety Problems (6 items, Cronbach's α = 0.75).

#### The early adolescent temperament questionnaire, revised

The Early Adolescent Temperament Questionnaire, Revised (Ellis and Rothbart, 2001) is a 62-item questionnaire that assesses temperamental and behavioral dimensions in adolescents aged 9-15. Items are rated on a 5-point Likert scale ranging from 1 (*almost always untrue*) to 5 (*almost always true*). The Negative Affect scale used in this study comprises a mean score calculated from scores on temperamental subscales Frustration (6 items), Shyness (5 items), Fear (6 items) and behavioral subscales Aggression (7 items) and Depressive Mood (5 items), reflecting negative emotional reactivity and lability as well as depressive and fearful mood states (Snyder et al., 2015). The scale Effortful Control consists of the subscales Attention (6 items), Inhibitory Control (5 items) and Activation Control (7 items), assessing frontally-mediated self-regulatory capacities. There was no Norwegian translation of the EATQ-R for the 9 to 15-year-old age group so two of the authors performed a translation which was then back-translated by an independent translator and finally approved by one of the original creators of the EATQ-R (L. K. Ellis, personal communication, June 2^*nd*^, 2014). The Cronbach's α for the different subscales in the translated version of the EATQ-R were comparable to the original version; Aggression: 0.80 (original scale 0.79); Depressive Mood: 0.76 (0.74); Frustration: 0.84 (0.83); Fear: 0.71 (0.69); Shyness 0.87 (0.72); Activation Control 0.88 (0.66); Attention 0.75 (0.65); and Inhibitory Control 0.65 (0.86).

Whether mothers or fathers completed the CBCL and the EATQ-R varied between families and therefore the parental ratings represent ratings from both mothers and fathers. Parental ratings have been shown to be generally consistent, but as some studies have shown that fathers are better at predicting future problems (Webster-Stratton, 1988; Hay et al., 1999) the paternal rating was chosen when both parents had responded.

#### Psychophysiological protocol

Before the psychophysiological recordings began all children were in the lab for a minimum of 30 min, the first 15 of which at least one parent was also present. The children and parents received a briefing about the equipment, the protocol and the right to terminate. Following this, parents were escorted to a private waiting area and briefed about the study questionnaires. The children were instructed to use the bathroom, put on a t-shirt, and remove their shoes. The children were weighed on a freestanding scale (Coline brand). Height was measured against a wall. The electrode sites were then lightly washed and scrubbed (NuPrep scrub gel). Three Ag/AgCl electrodes were attached in a lead-II ECG configuration on the clavicles and bottom rib. Four sets of double-Ag/AgCl electrodes were attached to the sides of the upper torso and neck for the ICG. A Biopac TSD201 respiratory belt was also fastened around the torso to monitor respiration. The last 10 min before the recording began the children were seated quietly in the experimental chair, while the experimenter readied the equipment. The children were instructed to sit as quietly as possible with their eyes open. The children were not instructed to focus on their breathing in any way. The recordings were controlled by a Cedrus Stimtracker, and were 300 s long.

The Biopac MP150 system with AcqKnowledge version 4.4 software and the ECG 100C amplifier was used for the ECG recordings. The sampling rate was set to 1,000 Hz, with a 50 Hz notch filter to control for power-line artifacts. A 1.0 Hz high pass and a 35 Hz low-pass filter was also applied to the ECG signal. The ICG was recorded with the MP150 system and a wireless Biopac Bionomadix transmitter. On acquisition a 10 Hz low-pass filter was applied to the ICG signal.

The recordings took place in Oslo and Trondheim, using the same equipment and the same protocol, conducted by female experimenters. The locations were set up to be as identical as possible, and were temperature controlled, quiet, and visually sparse. To rule out any confounding effects of location, location was included as a controlling variable in the generalized models. As the preliminary models did not show any signs that location was a significant variable, it was not included in the final models.

#### Biometric analysis

##### Pre-processing

For analysis of the five-min recording of ECG data HRV-software Kubios version 3.0.1 (Tarvainen et al., 2014) was used, with a 500-lambda smoothness prior filter to reduce the influence of very low frequency components and drift. The data was inspected visually for artifacts and misidentified beats were corrected manually in Kubios by shifting the indicator for the R-point, using the RR tachogram and the raw ECG signal to locate the correct point in the ECG-signal. Visually identified ectopic beats were corrected with interpolated RR-values using the automatic artifact correction option in Kubios. No additional beats were corrected beyond those already identified as artifacts. A further description of the Kubios artifact-correction procedure can be found in the Kubios manual (Tarvainen et al., 2017). To control for the mathematical impact of heart rate (HR) on HRV (Sacha, 2014), mean heart rate was extracted and used as a controlling variable in the generalized models.

##### root mean square of successive differences (RMSSD)

RMSSD is a much-used measure of vagally-mediated high-frequency heart rate variability (HF-HRV) that is highly correlated with high-frequency components of the heart signal (Massin et al., 1999). To ascertain the validity of this assumption in the current data set, RMSSD was correlated against a measure of absolute HF power in the RR-signal (HF power ms^2^, calculated by autoregressive modeling, 16 model-order) yielding a very strong positive relationship between RMSSD and HF ms^2^ in both groups (clinical *r* = 0.94, *p* ≤ 0.001; control *r* = 0.95, *p* ≤ 0.001).

##### Calculation of beat-to-beat instantaneous HR and RMSSD

To allow for investigation of possible group, age or gender distinctions in the development of HR and RMSSD across the 5 min of baseline, instantaneous beat-to-beat HR and RMSSD was calculated from the interbeat-interval in R (R Core Team, 2017) using the “varian” package (Wiley and Elkhart Group Limited, 2016). RMSSD calculated from ultrashort 30-s segments have shown good correspondence with longer recordings (Munoz et al., 2015), so 30-s segments were chosen. For each 30-s interval the mean HR and RMSSD for each subject was calculated.

##### sample entropy (SampEn)

SampEn (Richman and Moorman, 2000; Lake et al., 2002) calculates the conditional probability that two pattern sequences of *m* number of consecutive points that match within a tolerance *r*, still match when one additional consecutive point is included. High values of SampEn indicate high irregularity and complexity in the signal. SampEn was calculated with a tolerance (*r*) of 0.2 standard deviation of the R-R interval and an embedding dimension (*m*) of 2.

##### detrended fluctuation analysis (DFA)

DFA was calculated on the original RR-interval time-series before the detrending filter was applied to avoid detrending twice. The DFA algorithm is a modified root mean square analysis of a random walk (Peng et al., 1995). DFA is used to detect long-term correlations in a non-stationary time series, and calculates the correlation within non-overlapping segments of the signal. In each segment a least-squares regression-line is formed, and a detrended time series is then generated by subtracting regression from the integrated series. The final fluctuation is calculated from the detrended and integrated series, giving a scaling exponent, α. For this study the short term scaling exponent, DFA-α_1_, was calculated with *n* varying from 4 to 16 beats, giving an estimate of short-term correlations in the signal. Within these parameters DFA-α_1_ will likely capture a mixture of both low- and high-frequency components. A short term scaling exponent of α_1_ of 0.5 is indicative of a random and uncorrelated signal resembling white noise while an α_1_ level between 0.5 and 1 is indicative of positive correlations and self-similarity in the signal. Conversely, a signal between 0 and 0.5 is indicative of negative correlations in the signal (Hardstone et al., 2012).

##### pre-ejection period (PEP)

PEP was analyzed in Mindware IMP 3.1.4, using the baseline impedance signal (*Z*_0_) and rate of change in impedance (dZ/dt). The signal was inspected visually for noise and noisy segments were excluded. PEP was identified based on the interval separating the R-onset/Q-peak in the ECG, and the B-point in the dZ/dt signal. The R/Q point was estimated using minimum value K-R interval (K value 35) (Berntson et al., 2004) and the B-point was estimated through percent of dZ/dt time (55 percent) (Lozano et al., 2007). An average PEP for five 60-s epochs was calculated using a scale factor of 0.09 V/Ω for *Z*_0_ and 1.0 V/Ω for dZ/dt.

#### statistical analysis

For statistical analysis SPSS version 25 (IBM) and R version 3.4.4 (R Project) was employed. Outliers in the psychophysiological and psychological data (± 3.29 SD) were removed. Between group-differences on the most important variables were checked with independent t-tests. Differences in the instantaneous HR and RMSSD were investigated using t-tests on individual means within each non-overlapping 30-s subinterval of the 5-min recording. Analysis was run checking for differences resulting from group-membership, age (by median split at 10.7 years) and sex.

Effect size for significant between group-differences is given as Cohen's *d*. The correlations between cardiac variables and the relationship between the non-linear HRV variables and the EATQ-R subscales were checked with Pearson product moment correlations. The Affective Problems and Anxiety Problems subscales were analyzed with non-parametric Spearman's rank-order correlations, due to a distribution skewed toward low problems in the control group. Strengths of associations are discussed as follows: a correlation coefficient of 0.30 to 0.50 indicates a weak relationship, 0.50 to 0.70 a moderate relationship, 0.70 to 0.90 a strong relationship and > 0.90 indicates a very strong relationship between variables.

Generalized linear modeling with a maximum likelihood estimate and link ID function was used for analysis of the relationships between the physiological variables and internalizing psychopathology and negative affect. Results are given as full model effects for all variables added to each model. For variables showing significance above 0.01 and 0.05 level, B (and 95 % confidence intervals) and Exp(B) are also given. The B-value represents the slope in the linear equation, and can be read as follows: when X (the independent variable) rises with 1, this will lead to an increase/decrease in Y (the dependent variable) that equals B. All independent variables were transformed to Z-scores for a more standardized interpretation of the B-values across variables. Exp(B) is an odds-ratio estimate of an outcome given a one unit increase in the independent variable, and gives an estimate of effect size. An Exp(B) above 1 indicates a likely increase in the dependent variable given an increase in the independent variable, while an Exp(B) of less than 1 indicates a likely decrease in the dependent variable given an increase in the independent variable.

Significance will be discussed at both a *p* ≤ 0.01 and a *p* ≤ 0.05 level to balance the risk of type 1 and type 2 errors due to sample size and the exploratory nature of the study. The results need to be interpreted with this in mind.

## Results

### Between-group differences

The clinical and control group differed significantly on the Internalizing Problems, Negative Affect and Effortful Control scales. The two groups did not differ significantly in regard to SampEn, DFA-α_1_, HR or RMSSD for the five-minute sample as a whole, but PEP was shorter in the control group than in the clinical group. Descriptive statistics and between group differences can be seen in Table 1. Analysis of instantaneous HR and RMSSD in 30-s segments across the 5 min showed no significant differences between the clinical subjects and the controls, males and females or younger and older subjects.

**Table 1:** Descriptive statistics and between-group differences.

	**Clinical group**			**Control group**			**Between-group differences**		
	***n***	***Mean***	***SD***	***n***	***Mean***	***SD***	***t***	***p***	***d***
Internalizing Prob.	32	6.4	0.8	25	4.4	0.8	9.1	≤0.001^***^	2.4
Affective Problems	32	6.4	0.9	25	5.1	0.3			
Anxiety Problems	32	6.4	0.7	25	5.1	0.3			
Negative Affect	32	2.7	0.5	25	2.0	0.5	4.8	≤0.001^***^	1.3
Aggression	32	2.2	0.7	25	1.7	0.6			
Frustration	32	2.9	0.8	25	2.4	0.7			
Depressive Mood	32	3.1	0.7	25	2.0	0.6			
Fear	32	2.6	0.6	25	2.1	0.7			
Shyness	32	2.9	0.9	25	2.3	0.6			
Effortful Control	32	3.4	0.6	25	3.8	0.5	−2.5	0.018^*^	−0.7
HR	32	78.2	8.3	25	80.3	8.8	−0.9	0.36	
PEP	30	122.3	6.1	25	117.9	5.8	2.7	0.009^**^	0.7
RMSSD	31	73.8	39.4	25	67.9	35.9	0.6	0.56	
SampEn	31	1.7	0.2	25	1.7	0.2	0.4	0.71	
DFA-α_1_	32	0.8	0.2	25	0.8	0.2	−0.3	0.77	

Between group differences was conducted using independent t-tests (t); SD, standard deviation; DFA, detrended fluctuation analysis; HR, mean heart rate; RMSSD, root mean square of successive differences; PEP, pre-ejection period; SampEn, sample entropy;

* p ≤ 0.05;

** p ≤ 0.01;

*** p ≤ 0.001, a minor portion of this table has been published in Fiskum et al. (2017)

### Correlations between HRV-variables

SampEn showed a weak negative correlation to HR in the control group, and no significant relationship to any other cardiac variables. DFA-α_1_ was moderately positively related to HR in the clinical group and moderately negatively related to RMSSD in both groups. RMSSD was also moderately negatively related to HR in both groups, while PEP showed a weak relationship to HR (negative) and RMSSD (positive) in the control group. See Tables 2, 3 for results.

**Table 2:** Correlations between cardiac variables in the clinical group.

		**MeanHR**	**PEP**	**RMSSD**	**SampEn**
MeanHR	*r*	1			
	*p*	–			
	*n*	32			
PEP	*r*	−0.32	1		
	*p*	0.08	–		
	*n*	30	30		
RMSSD	*r*	−0.61^***^	0.15	1	
	*p*	<0.001	0.43	–	
	*n*	31	29	31	
SampEn	*r*	−0.19	0.16	−0.25	1
	*p*	0.31	0.40	0.18	–
	*n*	31	29	30	31
DFA-α_1_	*r*	0.57^***^	−0.29	−0.65^***^	0.19
	*p*	0.001	0.13	<0.001	0.31
	*n*	32	30	31	31

r, Pearson product-moment correlation coefficient; DFA, detrended fluctuation analysis; RMSSD, root mean square of successive differences; PEP, pre-ejection period; SampEn, sample entropy;

*** *p ≤ 0.001*.

**Table 3:** Correlations between cardiac variables in the control group.

		**MeanHR**	**PEP**	**RMSSD**	**SampEn**
MeanHR	*r*	1			
	*p*	–			
	*n*	25			
PEP	*r*	−0.47^*^	1		
	*p*	0.02	–		
	*n*	25	25		
RMSSD	*r*	−0.68^***^	0.49^**^	1	
	*p*	< 0.001	0.01	–	
	*n*	25	25	25	
SampEn	*r*	−0.46^*^	0.04	0.15	1
	*p*	0.02	0.87	0.47	–
	*n*	25	25	25	25
DFA-α_1_	*r*	0.35	−0.33	−0.53^**^	−0.31
	*p*	0.09	0.10	0.01	0.13
	*n*	25	25	25	25

r, Pearson product-moment correlation coefficient; DFA, detrended fluctuation analysis; RMSSD, root mean square of successive differences; PEP, pre-ejection period; SampEn, sample entropy;

* p ≤ 0.05;

** p ≤ 0.01;

*** *p ≤ 0.001*.

### Correlations with the CBCL and EATQ-R-subscales

Further, we ran correlations between the non-linear cardiac variables and the CBCL subscales of Affective and Anxiety Problems. SampEn was weakly negatively related to Affective Problems at a ≤ 0.05 level and showed a nearly-significant trend toward a weak negative relationship with Anxiety Problems in the clinical group. For the control group there was a moderate negative correlation with Anxiety Problems at a ≤ 0.01 significance level. DFA-α_1_ did not show any significant relationships in either group. See Table 4 for results.

**Table 4:** Correlations between CBCL clinical scales and cardiac variables.

		**Affective Problems**	**Anxiety Problems**
**CLINICAL GROUP**			
SampEn	ρ	−0.41^*^	−0.35
	*p*	0.02	0.053
	*n*	31	31
DFA-α_1_	ρ	−0.16	−0.19
	*p*	0.38	0.31
	*n*	32	32
**Control Group**			
SampEn	ρ	0.06	−0.50^**^
	*p*	0.79	0.01
	*n*	25	25
DFA-α_1_	ρ	0.13	0.09
	*p*	0.55	0.68
	*n*	25	25

ρ, Spearman's ranked order correlation coefficient; DFA, detrended fluctuation analysis; SampEn, sample entropy;

* p ≤ 0.05;

** *p ≤ 0.01*.

We also ran correlations between the non-linear cardiac variables and the EATQ-R Negative Affect subscales (Aggression, Fear, Frustration, Depression and Shyness) as well as the Effortful Control scale. For the EATQ-R scales a ≤ 0.01 level significant negative relationship of moderate strength could be found between SampEn and the subscales Aggression, and Frustration in the clinical group. There was a weak positive relationship between SampEn and Effortful Control (*p* ≤ 0.05). There were no significant correlations in the control group. DFA-α_1_ was not significantly related to any of the subscales in either group. See Table 5 for results.

**Table 5:** Correlations between EATQ-R scales and cardiac variables.

		**Aggression**	**Frustration**	**Depressive Mood**	**Fear**	**Shyness**	**Effortful Control**
**CLINICAL GROUP**							
SampEn	*r*	−0.53^**^	−0.52^**^	−0.30	−0.28	−0.19	0.40^*^
	*p*	0.002	0.003	0.11	0.13	0.31	0.02
	*n*	31	31	31	31	31	31
DFA-α_1_	*r*	−0.09	−0.21	−0.11	−0.17	−0.03	−0.08
	*p*	0.61	0.25	0.57	0.36	0.88	0.67
	*n*	32	32	32	32	32	32
**CONTROL GROUP**							
SampEn	*r*	−0.18	−0.36	−0.04	−0.32	−0.15	0.13
	*p*	0.39	0.08	0.84	0.12	0.48	0.55
	*n*	25	25	25	25	25	25
DFA-α_1_	*r*	0.36	−0.01	−0.02	−0.22	0.22	0.004
	*p*	0.08	0.95	0.93	0.28	0.28	0.99
	*n*	25	25	25	25	25	25

r, Pearson product-moment correlation coefficient; DFA, detrended fluctuation analysis; SampEn, sample entropy;

* p ≤ 0.05;

** *p ≤ 0.01*.

### SampEn, DFA-α_1_, RMSSD and PEP as discriminators of internalizing psychopathology

We ran two generalized linear models with Internalizing Problems as the dependent variable, and SampEn (Model 1) and DFA-α_1_ (Model 2) as independent variables alongside RMSSD and PEP. All models also controlled for group, sex, age, BMI, HR and a group-interaction. Both models were significant, with no significant group-interactions.

The results from Model 1 showed that a higher SampEn was significantly associated with less psychopathology at a ≤ 0.01 confidence level. RMSSD and PEP did not reach significance.

The results from Model 2 showed that a higher value of DFA-α_1_ was related to less Internalizing Problems at a ≤ 0.05 confidence level. RMSSD and PEP were not significantly related to Internalizing Problems.

Model effects and effect sizes for both models can be seen in Table 6.

**Table 6:** Model effects for DFA-α_1_, SampEn, RMSSD, and PEP as predictors of internalizing psychopathology.

**Model effects for SampEn, RMSSD, and PEP as predictors of Internalizing Psychopathology**.										
						**95% CI**				
		***n***	***df***	**χ^2^**	***p*_χ^2^_**	**−95%**	**B**	**+95%**	**EXP(B)**	***p*_*B*_**
Full model		52	9	55.2	≤ 0.001^***^					
Factors	Group			56.9	≤ 0.001^***^	−2.4	−1.9	−1.4	0.2	≤ 0.001
	Sex			0	0.97					
Co-variables	Age			0.1	0.72					
	BMI			0.8	0.39					
	HR			0.6	0.46					
Predictors	RMSSD			1.5	0.23					
	PEP			0.0	0.93					
	SampEn			8.0	0.005^**^	−0.8	−0.4	−0.1	0.7	0.024
Interaction	Group × SampEn			0.3	0.56					
**Model effects for DFA-**α_1_**, RMSSD, and PEP as predictors of Internalizing Psychopathology**										
Full model		54	9	57.0	≤ 0.001^***^					
Factors	Group			80.8	**≤** 0.001^***^	−2.6	−2.1	−1.7	0.1	≤ 0.001
	Sex			1.3	0.26					
Co-variables	Age			0.3	0.56					
	BMI			0.0	0.99					
	HR			0.7	0.41					
Predictors	PEP			0.5	0.49					
	RMSSD			0.5	0.48					
	DFA-α_1_			4.3	0.039^*^	−0.9	−0.5	−0.1	0.6	0.010
Interaction	Group × DFA-α_1_			2.9	0.09					

Results were calculated with a generalized linear model using a normal probability distribution and identity link function. BMI, body mass index; HR, heart-rate; RMSSD, root mean square of successive differences; PEP, pre-ejection period; SampEn, sample entropy; DFA, detrended fluctuation analysis;

* p ≤ 0.05;

** p ≤ 0.01;

*** *p ≤ 0.001*.

### SampEn, DFA-α_1_, RMSSD and PEP as discriminators of negative affect

We ran two generalized linear models with Negative Affect as the dependent variable and SampEn (Model 3) and DFA-α_1_ (Model 4) as independent variables alongside RMSSD and PEP. All models also controlled for group, sex, age, BMI, HR and a group-interaction. Both models were significant, with no significant group-interactions.

The results from Model 3 showed that a higher value of SampEn was significantly related to less Negative Affect at a ≤ 0.01 level. RMSSD also reached significance in this model (*p* ≤ 0.05), with greater RMSSD associated with less Negative Affect.

For Model 4 DFA-α_1_ showed a trend toward significance at a ≤ 0.05 level, with a higher value being related to less Negative Affect. PEP reached significance (*p* ≤ 0.05), with a longer PEP associated with less Negative Affect.

Model effects and effect sizes for both models can be seen in Table 7.

**Table 7:** Model effects for DFA-α_1_, SampEn, RMSSD, and PEP as predictors of Negative Affect.

**Model effects for SampEn, RMSSD, and PEP as predictors of Negative Affect**										
						**95% CI**				
		***n***	***df***	**χ^2^**	***p*_χ^2^_**	**−95%**	**B**	**+95%**	**EXP(B)**	***p*_*B*_**
Full model		52	9	37.9	≤0.001^***^					
Factors	Group			34.1	≤0.001^***^	−1.0	−0.7	−0.5	0.5	≤ 0.001
	Sex			0.6	0.46					
Co-variables	Age			2.2	0.14					
	BMI			1.9	0.17					
	HR			1.6	0.21					
Predictors	RMSSD			5.3	0.021^*^	−0.5	−0.3	−0.04	0.8	0.021
	PEP			1.5	0.22					
	SampEn			8.9	0.003^**^	−0.5	−0.3	−0.1	0.8	0.005
Interaction	Group × SampEn			0.1	0.77					
**Model effects for DFA-**α_1_**, RMSSD, and PEP as predictors of Negative Affect**										
Full model		53	9	34.6	≤0.001^***^					
Factors	Group			39.8	≤0.001^***^	−1.0	−0.8	−0.6	0.5	≤ 0.001
	Sex			3.2	0.074					
Co-variables	Age			1.6	0.21					
	BMI			0.0	0.98					
	HR			0.5	0.50					
Predictors	PEP			4.0	0.045^*^	−0.3	−0.2	−0.003	0.9	0.045
	RMSSD			1.5	0.22					
	DFA-α_1_			3.1	0.08					
Interaction	Group × DFA-α_1_			1.8	0.18					

Results were calculated with a generalized linear model using a normal probability distribution and identity link function. BMI, body mass index; HR, heart-rate; RMSSD, root mean square of successive differences; PEP, pre-ejection period; SampEn, sample entropy; DFA, detrended fluctuation analysis;

* p ≤ 0.05;

** p ≤ 0.01;

*** *p ≤ 0.001*.

## Discussion

### Non-linear HRV, internalizing psychopathology and negative affect

Several studies have shown that internalizing psychopathology and dysregulated negative affect are associated with a loss of non-linear HRV in the heartbeat in adults (Kemp et al., 2010; de la Torre-Luque et al., 2016), but the relationship between non-linear HRV and psychopathology is not as well studied in children (Paniccia et al., 2017). Two main domains of methods have shown promise, the informational domain, including sample entropy (SampEn), and the invariant domain, including detrended fluctuation analysis (DFA). This study investigated SampEn and DFA-α_1_ in children related to internalizing psychopathology and negative affect. As HRV has been proposed as a biomarker of dysregulation and psychopathology (Beauchaine and Thayer, 2015; Kemp et al., 2017), and the neurovisceral integration model (Thayer and Lane, 2000, 2009) models psychopathology as a loss of vagal tone and resulting disinhibition of the SNS, we also wanted to see if non-linear methods could provide more information than a measure of vagally-mediated high-frequency HRV, the root mean square of successive differences (RMSSD) and a measure of SNS-mediated cardiac contractility, pre-ejection period (PEP).

The results showed that a higher value of SampEn was significantly associated with less internalizing psychopathology and negative affect when modeled alongside RMSSD and PEP. A higher DFA-α_1_ was also significantly related to less internalizing psychopathology, and showed a trend toward significance for negative affect, in the same direction. Neither RMSSD nor PEP were significantly related to internalizing psychopathology. Higher RMSSD showed a significant relationship to less negative affect when modeled alongside SampEn. A longer PEP, indicating less SNS activity, was related to less negative affect when modeled alongside DFA-α_1_. Correlational analysis between SampEn and DFA-α_1_ and the EATQ-R subscales related to internalizing psychopathology and negative affect showed that having a higher SampEn was linked to less aggression and frustration in the clinical group. There was also a positive relationship between higher SampEn and better effortful control, a protective factor against psychopathology linked to frontal function, in the clinical group. This links SampEn to established indices of frontal function and dysfunction (Bishop et al., 2004; Blair, 2004; Fekete et al., 2014). DFA-α_1_ was not significantly related to any of the subscales in either group.

### Between-group differences

There were no significant differences between the two groups in HR, RMSSD, SampEn or DFA-α_1_ for the 5-min recordings. The clinical group showed lower sympathetic activation, as reflected in a longer PEP, than the control group. In the neurovisceral integration model psychopathology is linked to increased SNS activation, so these results are contradictory to both theory (Thayer and Lane, 2000, 2009) and previous empirical findings linking shorter PEP (Light et al., 1998; Schweiger et al., 1998; Buss et al., 2004; Muñoz and Anastassiou-Hadjicharalambous, 2011) to internalizing problems in children and adults.

The lack of significant differences for the non-linear variables could mean that neither SampEn nor DFA-α_1_ may be sensitive enough to distinguish between subjects suffering from less-severe psychopathology and normal-functioning controls. The clinical group was not a high-psychopathology group, instead presenting with lighter clinical problems related to anxiety and depression. Following this there might have been differences between the control group and a more pathological group. The lack of difference in RMSSD is supportive of this hypothesis, as resting state recordings of RMSSD have been found to distinguish between healthy controls and children and adolescents with clinical problems (Koenig et al., 2016; Paniccia et al., 2017). It is also possible that the situation itself, which was a resting baseline without a specific stressor, was not sufficiently powerful to elicit reliable group differences related to autonomic stress responsivity. A reactive laboratory task or an ecological measurement of stressful activation in the environment would likely be able to yield more information on group differences. A more finely grained analysis of the non-linear measures as they evolved over a time-line might also have yielded further results.

Another important consideration is tied to the possibility of differences in multilevel complexity. Non-linear complexity, or loss thereof, may reveal itself on different timescales (Bornas et al., 2006), making a case for a multi-scaled analysis of non-linear complexity (Costa et al., 2002). A further multi-scale entropy analysis of the signal on different time scales could have revealed more information, and the same could have been the case for further investigation of different scales of resolution for DFA-α_1_, but this was beyond the scope of the current study.

### The nature of the relationship between cardiac complexity, internalizing psychopathology and negative affect: the optimum variability hypothesis

Taken together, the results from the generalized models and correlations showed a linear relationship between SampEn and internalizing psychopathology and negative affect. The correlational analyses were mostly significant in the clinical group, but the relationships were generally in the same direction across groups. The clinical sample showed higher levels of internalizing psychopathology and negative affect, which could account for the discrepancies in the strength of the associations in the correlations. A recent meta-analysis of the correspondence of vagally-mediated HRV measures to depressive problems in children also found non-significant correlational results for healthy controls (Koenig et al., 2016), indicating that the relationships may be less pronounced in healthy populations. Another possibility is that the relationship is quadratic, rather than linear.

It has been assumed that higher variability or complexity is linearly related to health (Guastello, 2015), and this has generally been supported by empirical findings related to psychopathology (de la Torre-Luque et al., 2016). However, the hypothesis of optimal variability posits a quadratic relationship, where optimal function resides in a band of optimal complexity, defined by high organization and high openness to input-driven variability (Guastello, 2015; Schuldberg, 2015). Outside of the range of optimal variability function will be affected negatively, due to either too much organizational constraint or too little stability. This quadratic relationship may present as linear in smaller samples, or in samples spanning the lightly-dysfunctional to the normally-functional. If this happens, relationships in the data may present as smaller than they would be, given a larger or more heterogenous population (Guastello, 2015). In this study, the relationship between non-linear HRV and internalizing psychopathology and negative affect presented as linear, with higher SampEn associated with less internalizing psychopathology and less negative affect and a higher DFA-α_1_ linked to less internalizing psychopathology. There was a stronger relationship in the clinical group than in the healthy controls, although group-status did not yield a significant interaction in any of the models. Following the optimal variability hypothesis, it could be that a larger, or differently-distributed sample could have revealed a different and perhaps more curvilinear relationship between the non-linear HRV-variables and psychopathology and negative affect, and that the weaker relationship in the healthy sample is indicative of a relationship that may actually be curvilinear in the population (Guastello, 2015).

### Implication of the results

In the neurovisceral integration model (Thayer and Lane, 2000, 2009) negative affect and psychopathology can be modeled as a loss of adaptability in the neurovisceral system characterized by a state of chronic frontal disinhibition and vagal withdrawal. This dysregulated state in turn leads to sympathetic hyperarousal and negative attentional, emotional, cognitive, behavioral and physiological consequences. The results in this study support that lower vagal and higher SNS activation were related to dysregulation of negative affect, although the clinical group showed lower SNS activation as indexed by PEP than the controls. The results also indicated that vagal and SNS activation were not the entire story, as the generalized models showed that SampEn and DFA-α_1_ were more sensitive indicators of internalizing psychopathology than vagally-mediated HF-HRV and sympathetic activation combined. SampEn also held explanatory power beyond that accounted for by RMSSD and PEP related to negative affect. This indicates that non-linear HRV can provide information beyond what is contained in linear indices of vagal and SNS activation. It is however harder to pinpoint what non-linear HRV indexes, in terms of physiological correlates.

To try to clarify this question we have to distinguish between the physiology underlying informational complexity, as indexed by SampEn, and the physiology potentially involved in invariant short term self-similarity, as indexed by DFA-α_1_. Both informational and short-term invariant complexity have been found to be comparable between children and young adults, with a decline in informational complexity after age 40 (Pikkujämsä et al., 1999). DFA-α_1_ has been shown to be unrelated to sympathetic norepinephrine spillover, a marker of sympathetic cardiac activation (Baumert et al., 2009). DFA-α_1_ has also been shown to be negatively related to RMSSD, and to increase after vagal blockade (Perkiomaki et al., 2002). In our study DFA-α_1_ was strongly negatively related to RMSSD, and showed no relation to PEP. This indicates a likely vagal influence on DFA-α_1_ in our study.

In regard to the potential functional significance of DFA-α_1_, one hypothesis is that invariant self-similarity in a signal represents a form of internal long-term “memory” of a system (West, 2014). While a simple thermostat with one set-point reacts only to real-time fluctuations in temperature, with no memory of earlier fluctuations, this is not the case for complex systems. Behavior in a complex system relates to multiple set-points, across different time-scales. As a consequence, instead of operating only on one time-scale, the system displays multi-stability across different time-scales. In an earlier publication one of the present authors investigated a link between the attentional orienting network, vagal control and non-linear scaling properties (Balle et al., 2015). In this study, deficits in the attentional network were accompanied by alterations in the scaling properties, or long-term “memory” of the cardiac system.

The self-similar properties of the cardiac system may also be relevant in relation to interoception (Vaitl, 1996), where bodily signals, through for instance vagally-mediated afferent baroreceptor firing (Garfinkel et al., 2013, 2015), represent an internal context important for perception (Dunn et al., 2010; Seth, 2013). It has been shown that the perception of frightening emotional stimulus can be enhanced at different points in the cardiac cycle related to systole and diastole (Garfinkel et al., 2014; Garfinkel and Critchley, 2016). It has also been shown that high accuracy in interoception facilitates the predictability of internal signals, possibly increasing resilience to the effect of stimulus presentation timing (Garfinkel et al., 2013). Furthermore, the inability to integrate and predictively utilize information from interoception may be a common factor underlying emotional dysregulation and anxiety (Seth, 2013), linked to the anterior cingulate cortex and the anterior insula, two areas that are important for a feeling of “self-in-context” (Seth et al., 2012; Seth, 2013). In our study a higher DFA-α_1_ was related to less internalizing psychopathology. It could be speculated that perhaps higher scale-free self-similarity in the cardiac signal not only indexes a more complex underlying organization of the neurocardiac system, but also represents a more predictable inner context, enhancing the potential for predictability in interoception. Following this, perhaps a higher potential for predictability in inner context can have a positive effect on emotional perception and processing and the experience of “self-in-context” relevant for mental health. This would need to be investigated further and remains purely hypothetical at this point.

With respect to the physiological correlates of SampEn, the informational complexity in a signal is thought to reflect on the complexity and flexibility of the underlying system (Bertuglia and Vaio, 2005). A low-entropy system behaves in a very repetitive manner (the rate of new information it generates is close to zero), and is therefore less capable of adapting to the changing environment than a high-entropy system. Healthy hearts do not beat at a totally constant rate, but show many fluctuations that make it difficult to predict the length of the next interbeat interval. At the same time this increases their adaptability. Reduced cardiac entropy has been found to be related to vagal withdrawal and increased SNS-activation in anxious patients (see for instance de la Torre-Luque et al., 2016), with some exceptions linked to higher irregularity (and hence entropy) of respiration in panic-disordered patients (see for instance Caldirola et al., 2004). Evidence from vagal blockade (Millar et al., 2010) and norepinephrine spillover (Baumert et al., 2009) point to a likely higher influence of vagal than sympathetic activation in entropy rate. At the same time, investigation has also failed to find an effect of vagal blockade along with no correlations between entropy and cardiac measures of vagal origin (Perkiomaki et al., 2002). In our study SampEn was not significantly correlated with either RMSSD or PEP, but showed a weak negative relationship to heart rate in the controls. When RMSSD and PEP were modeled together as independent variables affecting SampEn, (as reported in Fiskum et al., 2017), lower RMSSD and higher PEP were linked to greater entropy. This indicates that SampEn did not relate to either vagal or SNS influence in a simple one-to-one linear way. From a theoretical point of view, non-linear cardiac complexity should indeed reflect on more emergent characteristics of whole-system behavior, than simpler, linear metrics (Bertuglia and Vaio, 2005; Tyukin, 2011; West, 2014).

From a more functional perspective, reduced SampEn has been linked to reduced flexibility in the neurovisceral system in adolescents at risk for anxiety disorder (Balle et al., 2013). A hypothesis in line with this is that non-linear HRV represents an index of the flexibility of the organization of the neurocardiac system, linked to underlying brain connectivity (Young and Benton, 2015). It is interesting to note that HF-HRV has been linked to higher local connectivity in the perigenual anterior cingulate cortex, a medial prefrontal area serving as a connective hub between the salience and default mode networks (Jennings et al., 2016). Resting HF-HRV has also been linked to stronger functional connectivity between amygdala and the medial-prefrontal cortex (Sakaki et al., 2016). The analysis of the subscales in this study linked levels of SampEn with markers of frontal and limbic function. Anxiety, aggression and frustration have been linked to diminished frontal functionality (Bishop et al., 2004; Blair, 2004) and greater amygdala reactivity (Rauch et al., 2003), while better effortful control has been linked to greater frontal functionality and connectivity (Fekete et al., 2014). Taken together, the results in this study show that SampEn may have potential as an index of the organizational flexibility and adaptability of the neurovisceral system, relevant to psychopathology and negative affect, that in turn may be linked to the organization of frontal and limbic areas of the brain.

## Conclusion

Overall, the results from this study were in line with the neurovisceral integration model in that lower SNS and higher vagal activation were related to less dysregulated negative affect. In line with the initial hypotheses information-based complexity, as indexed by SampEn, explained variation in negative affect beyond the combination of PNS/SNS activation. The results also showed that SampEn and DFA-α_1_ were the only variables significantly related to internalizing psychopathology, apart from group status.

This means that non-linear HRV provided information about the operation of the neurovisceral system relevant to internalizing psychopathology and negative affect beyond that of indices of parasympathetic and sympathetic activation combined. It has been suggested that HRV can be a measure of autonomic flexibility (Lipsitz, 2004; Beauchaine and Thayer, 2015), with low variability indexing a rigid autonomic balance, that is, a system that is less capable of flexible interaction with the outside environment. We propose that non-linear HRV holds potential as a complementary way to index adaptability related to the organization of the underlying neurovisceral system. Specifically, we suggest that cardiac entropy may be an indicator of the flexibility of the neurovisceral system underlying emotional regulation and adaptive behavior, that may be linked to integrated functionality or connectivity in frontal and limbic areas. We also propose a hypothesis that invariant self-similarity may influence a form of bottom-up predictability involved in interoception (or inner context), that may serve as a protective influence in regard to psychopathology.

### limitations and future research

The sample size was small, and this means that small- and moderate-effect-size relationships may have been overlooked (Quintana, 2017). Also, this study focused on internalizing psychopathology and negative affect, and did not investigate disorder across the externalizing or thought-disorder spectrum, which may have yielded further results. Another limitation is that the clinical group was not a high-psychopathology group. The clinical subjects were referred for lighter problems of anxiety and depression, meaning that the results may have presented differently in a more pathological group. The age-distribution of the sample also meant that the children may have been in different stages of puberty. Non-linear HRV in children between 6 and 15 years have been shown to not differ significantly from young adults, meaning that the measures of non-linear HRV should still be valid (Pikkujämsä et al., 1999). A further limitation is the lack of child-report of psychopathology and negative affect. To make up for this, the parent-report tools used were chosen for extensive clinical experience and use. The CBCL has also shown good correspondence between parent ratings and clinical judgment of psychopathology by professionals (Verhulst and van der Ende, 1991) and diagnostic criteria derived from the Diagnostic and Statistical Manual of Mental Disorders (DSMuncorrelated signal resembling) (Ebesutani et al., 2010).

Furthermore, it is a limitation that no autonomic stress maneuver designed to elicit sympathetic activation or a measure of sympathetic variability was employed. PEP can be influenced by several cardiovascular confounders including left ventricular end-diastolic pressure, systemic vascular resistance and diastolic blood pressure (Krohova et al., 2017), and none of these factors were controlled for. The inclusion of QT-variability, an index of spontaneous beat-to-beat fluctuations in the QT-interval indexing cardiac repolarization lability (Baumert et al., 2016), would have strengthened the results. QT-variability has been linked to internalizing spectrum psychopathology (Yeragani et al., 2003; Koschke et al., 2009), also alongside differences in non-linear HRV (Yeragani et al., 2001) and should be considered as a focal measure of sympathetic variability in future studies. A suitable stress maneuver should also be included. The lack of control for respiration is a further limitation, as respiratory patterns can affect HRV.

Finally, the physiological correlates of non-linear HRV are not easy to pin down, and the relationships to psychopathology and health are not necessarily straightforward, so more research remains to show the clinical usefulness. While much research on HRV and emotional dysregulation is based on the analysis of resting baseline HRV-data, studies looking at non-linear HRV-data during the completion of stressful or negatively loaded emotional tasks would likely give additional information about the relationship between complexity, state and dysregulation. The results should be further explored in both younger and older populations, in community-based samples, and in other clinical populations as there may be differences across age- and patient-groups. One line of investigation that could potentially increase clinical usefulness would be the study of non-linear HRV-indices related to characteristics of brain-network connectedness in the neurovisceral system. Also, further research into invariant cardiac self-similarity as a potential moderator of the predictability of interoception, and possible effects on psychopathology and emotional dysregulation should be conducted.

## Author contributions

CF contributed to the original project formulation, conducted data collection, linear and non-linear HRV-analysis, statistical analysis and wrote the paper. TA contributed to the original project formulation, conducted data collection, PEP analysis, contributed on linear HRV-analysis and critically reviewed the paper. XB supervised and gave input on non-linear analysis and critically reviewed the paper. PA supervised and gave input on statistical analysis and critically reviewed the paper. KJ and MF supervised and contributed on the original project formulation and critically reviewed the paper.

### Conflict of interest statement

The authors declare that the research was conducted in the absence of any commercial or financial relationships that could be construed as a potential conflict of interest.
